# Tissue-specific autophagy responses to aging and stress in *C. elegans*

**DOI:** 10.18632/aging.100765

**Published:** 2015-06-30

**Authors:** Hannah C. Chapin, Megan Okada, Alexey J. Merz, Dana L. Miller

**Affiliations:** ^1^ Department of Biochemistry, University of Washington, Seattle, WA 98195, USA

**Keywords:** autophagy, aging, assay, target of rapamycin

## Abstract

Cellular function relies on a balance between protein synthesis and breakdown. Macromolecular breakdown through autophagy is broadly required for cellular and tissue development, function, and recovery from stress. While *Caenorhabditis elegans* is frequently used to explore cellular responses to development and stress, the most common assays for autophagy in this system lack tissue-level resolution. Different tissues within an organism have unique functional characteristics and likely vary in their reliance on autophagy under different conditions. To generate a tissue-specific map of autophagy in *C. elegans* we used a dual fluorescent protein (dFP) tag that releases monomeric fluorescent protein (mFP) upon arrival at the lysosome. Tissue-specific expression of dFP::LGG-1 revealed autophagic flux in all tissues, but mFP accumulation was most dramatic in the intestine. We also observed variable responses to stress: starvation increased autophagic mFP release in all tissues, whereas anoxia primarily increased intestinal autophagic flux. We observed autophagic flux with tagged LGG-1, LGG-2, and two autophagic cargo reporters: a soluble cytoplasmic protein, and mitochondrial TOMM-7. Finally, an increase in mFP in older worms was consistent with an age-dependent shift in proteostasis. These novel measures of autophagic flux in *C. elegans* reveal heterogeneity in autophagic response across tissues during stress and aging.

## INTRODUCTION

As cells respond to the varied demands of development, age, and stress they must actively curate a steady-state population of functional proteins and organelles. Disruption of the network that maintains this protein homeostasis (proteostasis) leads to cellular dysfunction and causes or aggravates pathologies associated with aging and disease [1–4]. Autophagy, or “self-eating,” helps cells maintain proteostasis by degrading dysfunctional cytoplasmic proteins and organelles. In times of stress, autophagic flux can also provide amino acids for energy and protein synthesis. A significant challenge is to understand how cellular autophagy influences the health and physiological responses of the organism as a whole. The model system *C. elegans* has yielded significant insights into the overall physiology of development, stress and aging, but it has been more challenging to understand the tissue-level nuances of autophagy's role in these processes.

Autophagy is a key downstream target of a central regulator of cellular growth and proteostasis, the Target of Rapamycin Complex 1 (TORC1). The TORC1 signaling network balances cellular growth against energy conservation and catabolism in response to changes in the availability of nutrients, energy, growth signals, and oxygen, as well as cellular stress [5–9]. Autophagy plays dual roles in TORC1-mediated proteostatic responses. First, autophagy removes proteins damaged by cellular stress, dysfunctional mitochondria, or proteins and structures with temporally-specific roles during development [10–14]. The second set of effects are even more broad: amino acids released during degradation help cells survive periods of starvation, and the products of autophagy also influence metabolism, membrane traffic, and immune responses [15–17]. Given these myriad effects, it is no surprise that both excessive and insufficient autophagy have been associated with the negative effects of disease, developmental defects, neuro-degeneration, and aging [3, 4, 15, 16, 18, 19].

Quantifying autophagy's magnitude in normal, autophagy-proficient tissues is challenging, and there are multiple assays that try to measure autophagic activity and flux (as reviewed by Klionsky: 20, 21). One reason autophagy is challenging to assay is that it is a multistep process. First, a portion of the cytoplasm is engulfed in a double-membrane-enclosed organelle termed the autophagosome. Fusion of the autophagosome with the lysosome exposes the cargo to attack by lysosomal hydrolases, degrading the autophagosome's contents. Two commonly-employed autophagy assays rely on the homologs of yeast ATG8/mammalian LC3, an orthologue of which in *C. elegans* is LGG-1, a member of the GABARAP/GATE-16 family. LGG-1 facilitates the growth of the autophagosome and is incorporated into its expanding membrane. One widely-deployed assay of autophagic initiation quantifies the accumulation of LGG-1-positive autophagosomes as fluorescent punctae. This assay has been used to reveal an increase in autophagy in food-deprived mice and worms [22–24], but is moderately labor-intensive, potentially difficult to interpret, and in *C. elegans* is limited to use in young nematodes due to age-dependent changes in cellular morphology and background autofluorescent punctae [25–27]. When the autophagosome and lysosome fuse in the final step of autophagy, this exposes a fraction of the LGG-1 to the acidic, proteolytic lumen of the lysosome. A second quantification method monitors the fusion of the autophagosome with the lysosome by using protein tags that change spectral properties or molecular mass when exposed to lysosomal pH and proteolysis [20, 28, 29]. This approach has been used in yeast, mammals and plants, but has not been widely used in nematodes [30]. The majority of the assays available for use in *C. elegans* rely on fluorescent assays and have significant caveats to their use and interpretation, suggesting that significant insight could come from a more accessible assay [31].

Studies in *C. elegans* have highlighted that autophagy is present in most tissues and has significant effects on longevity and metabolism [32]. However, there has not yet been a systematic survey of autophagic response across multiple tissues in the nematode. This leaves open the questions of when, where, and to what extent autophagy plays into organismal responses. To address these questions we created and validated a dual-fluorescent protein tag that generates a tissue-specific autophagy signal, detectable by immunoblot. This assay reveals that tissues in *C. elegans* vary both in the amount of basal autophagy and in the degree to which autophagy increases in response to different stresses. This first developmental map of autophagy in nematodes fundamentally shifts our understanding of autophagy and TOR-dependent responses in a premier model organism.

## RESULTS

### A new assay for measuring autophagy in *C. elegans*

We developed an immunoblot-based assay of autophagy for *C. elegans*. Similar assays of autophagy are commonly used in yeast, plants, and mammals [20, 33–36]. In nematodes, autophagy measurement generally favors counting GFP::LGG-1 punctae or other fluorescent assays, with few instances of monitoring substrate cleavage [37, 38]. As further discussed below, we sought to overcome the substantial limitations of fluorescent punctum-based autophagy measurements in *C. elegans* by developing a biochemical assay of autophagy. Our goals were to facilitate tissue-specific quantifications of autophagy, develop an assay that would also be usable in aged as well as young worms and provide a validated, two-part tag that could be used to measure autophagic degradation of either bulk cytoplasm or specific organelles.

**Figure 1:**
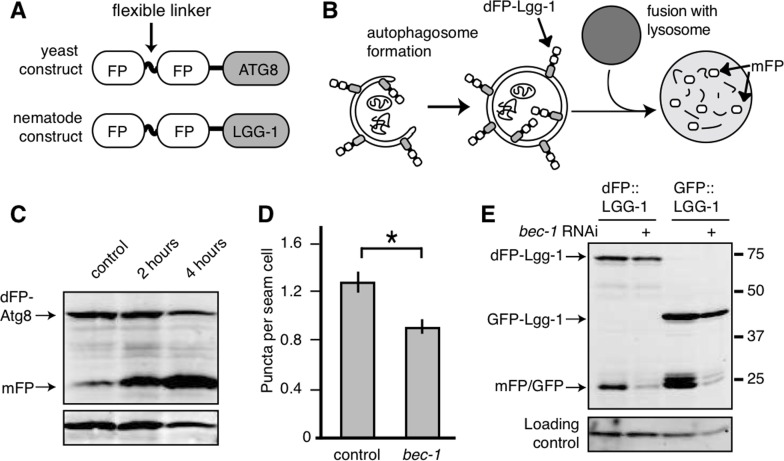
Monomeric FP released from dFP::LGG-1 correlates with autophagic flux (**A**) Yeast and nematode constructs were made by fusing a double fluorescent protein module, dFP, to ATG8 (yeast) or LGG-1 (nematode). The dFP contained two fluorescent proteins (labeled FP) separated by a protease-sensitive flexible linker. (**B**) The dFP Atg8/LGG-1 constructs are recruited to the growing autophagosome. Upon fusion of the autophagosome with the lysosome, the dFP is cleaved by lysosomal proteases, releasing soluble FP monomers. These monomers are fairly stable, but will eventually be further degraded by proteaolysis in the lysosome. (**C**) *S. cerevisiae* expressing dFP::ATG8 showed a time-dependent increase in mFP abundance with 2 or 4 hours of nitrogen starvation. (**D**) The number of dFP punctae in seam cells of L3 nematodes expressing dFP::LGG-1 from extrachromosomal arrays was reduced when the nematodes were treated with *bec-1* RNAi to impair autophagy, as has been previously observed for GFP::LGG-1 [14]. Error bars represent standard error, asterisk for p<0.05. (**E**) *C. elegans* with extrachromosomal arrays expressing either *eft-3*p::dFP::LGG-1 or *lgg-1*p::GFP::LGG-1 show mFP release. Full-length dFP::LGG-1 is present at 73kDa, GFP-LGG-1 at 40kDa, and the monomers of GFP or mFP are present at 25kDa. The accumulation of mFP was inhibited by treatment with *bec-1* RNAi (lanes 2 and 4). The accumulation of mFP in animals grown on control RNAi food is shown in lanes 1 and 3.

We tagged *C. elegans* LGG-1 with two fluorescent proteins connected by a flexible, protease-sensitive linker (dFP: dual Fluorescent Protein tag; Figure 1A). LGG-1 is incorporated into the growing autophagosome and the final step of autophagy, fusion of the lysosome and autophagosome, exposes LGG-1 to the lysosomal proteases. These proteases cleave the linker between the fluorescent tags, releasing monomeric FP (mFP) that is tightly folded and resistant to proteases (Figure 1B). An immunoblot probed with anti-FP antibody distinguishes between the full-length dFP::LGG-1 and the released mFP by size. Increased flux through the autophagic pathway results in an increase in the ratio of mFP to intact dFP::LGG-1. Therefore, this assay allows us to measure the flux through the autophagic pathway instead of the formation of autophagosomes that are marked with GFP::LGG-1. Importantly, this is also internally controlled, relying on the ratio of cleaved to uncleaved protein; this allows it to be used in comparisons between conditions with varying levels of protein expression. To verify that the dFP tag releases free mFP as a consequence of conditions known to increase autophagy we first validated the dFP tag in *Saccharomyces cerevisiae* cells using the LGG-1 homolog Atg8. When yeast expressing dFP-Atg8 were subjected to nitrogen starvation there was a time-dependent increase in mFP, showing that conditions that increased TOR-regulated autophagy also increased mFP. (Figure 1C). A quantification of the dFP and mFP bands, as well as the ratio between them, is shown for this and all other figures in Supplementary Table 1.

Next we sought to validate dFP::LGG-1 in nematodes. The most widely-used method for monitoring autophagy in *C. elegans* relies on counting GFP::LGG-1 punctae [8, 16, 24]. We validated our dFP::LGG-1 reporter by comparing it to this generally accepted system. Autophagy requires *bec-1,* the nematode orthologue of beclin/Vps30 [14, 20, 24]. We found that the number of fluorescent dFP::LGG-1 punctae in the seam cells of third-stage (L3) larvae decreased when *bec-1* was depleted by RNAi (Figure 1D), just as has been shown previously using the GFP::LGG-1 reporter [14, 24]. This result indicates that the dFP tag is effectively incorporated into growing autophagosomes.

We next wanted to validate that mFP release from the dFP reporter was also dependent upon autophagy. To do this we depleted *bec-1* by RNAi in animals ubiquitously expressing dFP::LGG-1. We found that *bec-1* RNAi reduced the amount of mFP released at steady-state from dFP::LGG-1 and GFP::LGG-1, just as we had expected (Figure 1E). We also observed a band consistent with cleavage of GFP from the GFP::LGG-1 fusion protein that was similarly sensitive to *bec-1(RNAi)*. Importantly, *bec-1* RNAi did not significantly affect the amount of full-length protein and only decreased the amount of cleaved (mFP or GFP) protein. Together, these results from punctae and immunoblot assay validate our dFP autophagy reporter.

### Autophagy varies substantially between tissues

**Figure 2:**
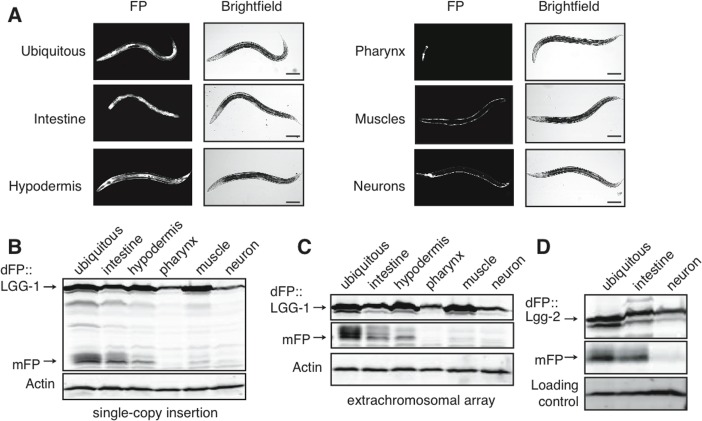
Tissue-specific expression of dFP::LGG-1 reveals variance in basal autophagy (**A**) *C. elegans* strains were created with dFP::LGG-1 expressed under control of a promoter that is ubiquitously active (*eft-3*p), or under the control of promoters driving expression specifically in the intestine (*vha-6*p), hypodermis (*dpy-7*p), muscles (*myo-3*p), pharynx (*myo-2*p), or neurons (*rab-3*p). An image of each strain shows fluorescence consistent with the expected tissue-specific expression. Animals were imaged at approximately late L3 larval stage; scale bar is 100mm. Visualizing the fluorescent protein using an anti-FP immunoblot revealed expression in mixed-age populations of strains with the reporter present in either (**B**) single-copy chromosomal insertion or (**C**) tandem, multi-copy extrachromosomal array. In both expression conditions mFP is most abundant in the intestine with some mFP present in the hypodermis. A full blot is shown in (**B**) to provide context, while subsequent images focus on the upper and lower bands of interest; uncropped blots are shown in Supplementary Figure 1. (**D**) dFP::LGG-2 expressed from the same ubiquitous, intestinal, or neuronal promoter showed lysosomal processing primarily in the intestines.

To measure autophagy in different tissues, we constructed strains with dFP::LGG-1 expressed under the control of tissue-specific promoters. These drove expression specifically in the intestines, hypodermis, muscles, pharynx, or neurons (Figure 2A). Tissue-specific lines were created using both high-copy extrachromosomal arrays and single-copy insertion into the genome. We assayed mixed-age populations of each strain by immunoblot and found that the intestines had the highest relative abundance of the mFP cleavage product. This was the case whether worms carried a single copy integrant (Figure 2B) or an extra-chromosomal array (Figure 2C, uncropped immunoblot for this and all other blots shown in Supplementary Figure 1) of the dFP::LGG-1 transgene. Importantly, we could not detect any experimental differences between the behavior of dFP::LGG-1 at low and high levels of expression in this assay, and quantifying the ratio of mFP to dFP in each tissue yielded relatively similar values independent of the amount of overexpression (Supplementary Table 1). Subsequent experiments employed animals expressing dFP reporters from transgene arrays.

LGG-1 is one of two *C. elegans* orthologues of mammalian LC3. Given the possibility of isoform-specific function [39–41], we asked whether the other LC3 orthologue LGG-2 might control different aspects of autophagic flux. Both proteins are broadly expressed, with largely overlapping expression patterns. There is slightly more LGG-2 present in neurons, raising the possibility of tissue-specific preferences of isoform incorporation into autophagosomes [42]. We therefore created a dFP::LGG-2 construct to measure autophagy that involves this protein. We found that dFP::LGG-2 had a tissue-specific signal of autophagic flux indistinguishable from that of dFP::LGG-1. Specifically, expression of full-length dFP::LGG-2 was detectable in all tissues, but dFP::LGG-2 released mFP primarily in the intestines and at very low levels in the neurons (Figure 2D). Thus, it appears that tissue-specific differences in mFP release reflect cell type-specific differences in the overall rate of autophagic flux, rather than reflecting functional variations between the two LGG proteins in *C. elegans*.

**Figure 3:**
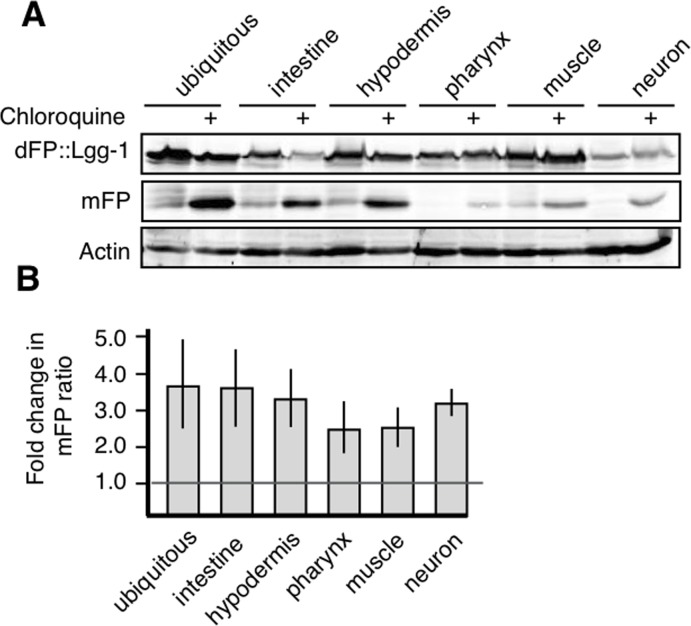
All tissues have active autophagic flux (**A**) Synchronized populations of L4 animals exposed to chloroquine for 18 hours showed accumulation of mFP in all tissues. (**B**) Average fold change over three experiments showing that all tissues increase mFP with chloroquine treatment, as shown by a fold change of greater than one, as marked by the horizontal line. Error bars represent standard error, tissue-specific chloroquine responses are not significantly different from each other (p>0.2).

The amount of mFP present at steady-state is a function of both autophagosome maturation (which would release mFP) and lysosomal degradation (which would remove mFP). We used chloroquine treatment to address the possibility that the accumulation or absence of mFP reflects the efficiency of lysosomal proteolysis rather than autophagosome maturation [21]. Chloroquine accumulates in the lysosome and prevents acidification, impairing protease function. We reasoned that if mFP accumulated in animals treated with chloroquine, this would confirm that mFP was released from dFP in the mature autophagosome as well as indicate that an absence of mFP at steady-state was due to efficient degradation of mFP by lysosomal proteases. We exposed synchronized populations of L4 nematodes to chloroquine and measured the release of mFP in different tissues. Chloroquine treatment caused an increase in mFP in all tissues. This increase is visible in the gel (Figure 3A) and quantification of the increase is also provided (Supplemental Table 1). Chloroquine treatment increased the ratio of mFP:dFP 1.5-fold or more above the control baseline in every tissue assayed. There was variation in the magnitude of the effect observed using this assay, but this effect was robust and readily replicated across tissues (Figure 3B). These data demonstrate that autophagy occurs in all tissues, but that mFP may be cleared from the lysosome too rapidly to be detected at steady-state in some tissues, such as neurons. We conclude that, as expected, the steady-state mFP levels we observe are influenced both by forward movement through the autophagic pathway and the activity of hydrolytic enzymes in the lysosome.

### Tissue-specific upregulation of autophagy in response to stress

**Figure 4:**
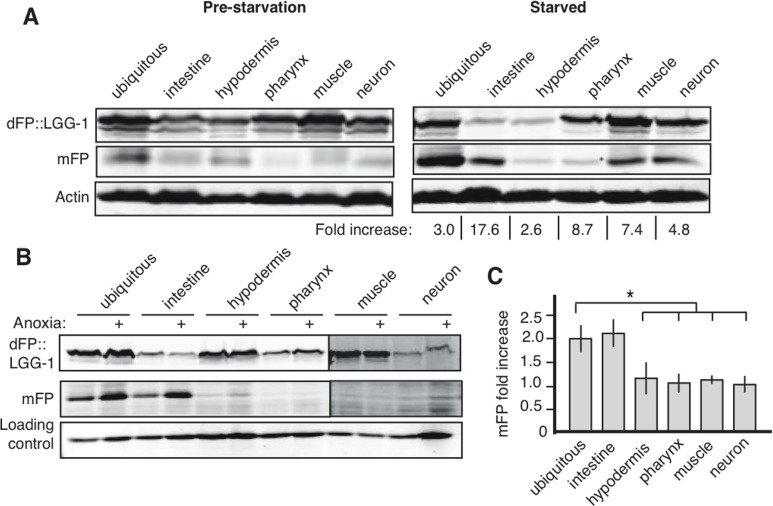
Starvation and anoxia induce different patterns of autophagy upregulation (**A**) Animals lysed at L1, before starvation, showed low levels of basal autophagy (left panel, and band intensities quantified in Supplementary Table 1). After 5 days of starvation there was an increase in mFP in all tissues (right panel). Fold increase indicates change in normalized mFP after starvation. Representative gel is shown but experiments consistently showed the largest increases in intestine, muscles and neurons, with a positive but smaller fold increase in other tissues. (**B**) Exposing nematodes to 18 hours of anoxia at mid-larval stage (L2/L3) causes an increase in autophagic flux in the intestine but does not affect mFP levels in other tissues. All lanes are from the same experiment run in the same SDS-PAGE gel, with delineations indicating areas of the image that were separately adjusted for brightness and contrast for the sake of clarity (uncropped immunoblots shown in Supplementary Figure 1). (**C**) A graph of the average fold change of normalized mFP in each tissue after anoxia. N=3-7, asterisk indicates p<0.05 for the mFP ratio of the ubiquitous reporter compared to the mFP ratio in the hypodermis, pharnynx or muscle, and p<0.06 for a comparison of the mFP ratios in ubiquitous and neuronal expression. The ubiquitous and intestinal reporters were statistically similar (p=0.8).

Autophagy is a key physiologic response to starvation, and the number of GFP::LGG-1 punctae increases in nematodes that receive no or very little food [16, 24]. Microscopic assays focus on only a readily-observed subset of cells within a starved organism, however, and studies rarely attempt to compare the effects of autophagy across multiple tissues in response to nutrient limitation. To address whether tissues differ in their upregulation of autophagy following starvation, we analyzed autophagy in animals that had been removed from food for five days. We observed at least a two-fold up-regulation in the relative amount of mFP, as compared to full-length dFP::LGG-1, in every tissue after starvation (Figure 4A). The change in flux was most dramatic in the intestines, but all tissues responded to starvation with an increase in autophagic flux. These results show for the first time that the regulation of autophagy during starvation is similar across all tissues, and is not specific to the minor subset of cells and tissues used for visualization of GFP::LGG-1 punctae.

Oxygen deprivation (anoxia; operationally defined as < 10 ppm O_2_) is similar to starvation in that cellular energy generation is severely limited. Moreover, as has been demonstrated for starvation, autophagy is required for nematodes to survive an anoxic shock [8]. We used our dFP::LGG-1 reporter to reveal the effect of anoxia on autophagic flux in different tissues. After 18 hours of anoxia, we observed an increase in mFP release that was primarily due to changes in the intestinal mFP accumulation (Figure 4B). In contrast, the ratio of mFP to full-length dFP::LGG-1 remained constant in other tissues of animals exposed to anoxia (Figure 4C). We conclude that, in marked contrast to the more general response to starvation, anoxia induces tissue-selective upregulation of autophagy in the intestine.

### Measuring autophagy of soluble cytoplasmic proteins

A potential confounding factor in experiments based on measuring GFP::LGG-1 is that overexpressing LC3 is reported to cause abnormal autophagosome formation and pathway dynamics [25]. We observed similar measurements of autophagic flux whether we expressed dFP::LGG-1 from multi-copy extrachromosomal arrays as when the reporter was expressed at a more natural level from single-copy integration into the genome (Figure 2), arguing that overexpression of dFP::LGG-1 was neither inhibitory nor stimulatory in our system. However, to circumvent this potentially confounding effect of LGG protein overexpression, we expressed dFP alone as a soluble cytoplasmic protein, without fusing it to any proteins directly involved in autophagy. We reasoned that mFP would be released from any of the cytoplasmic dFP taken into autophagosomes during bulk autophagic turnover, but that expressing mFP alone should not affect the formation of auto-phagosomes.

**Figure 5:**
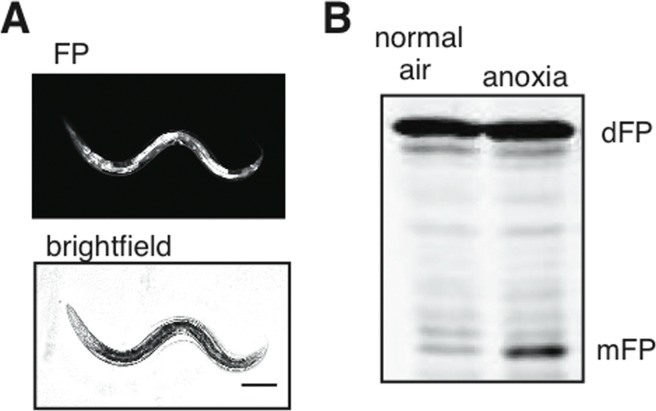
Cytoplasmic flux through the autophagic pathway (**A**) Ubiquitously-expressed soluble dFP was present in all tissues, as visualized with fluorescence. Scale bar 100mm. (**B**) This reporter showed a substantial increase in mFP release when the nematodes were exposed to anoxia. This demonstrates an increase of bulk autophagic flux without overexpression of any protein involved in autophagy.

The dFP construct driven by an *eft-3* promoter was expressed in all tissues, as expected (Figure 5A). We observed release of mFP in animals that expressed the soluble dFP reporter, consistent with our hypothesis that it would be taken up into autophagosomes non-specifically (Figure 5B). The release of mFP was enhanced by exposure to anoxia (Figure 5B), similar to our observation of increased mFP release from dFP::LGG-1 (Figure 4B). This result underscores that autophagic flux is induced in response to anoxia. The result further indicates that the dFP::LGG-1 fusion protein does not interfere with the assembly or maturation of autophagosomes.

### dFP reporters to visualize mitophagy

Our observation that mFP is released from solubly-expressed dFP suggests that this tag could be used to measure autophagy of specific proteins or organelles, even if the tagged protein or signal sequence is very small. This is in contrast to monomeric GFP, which cannot be used alone or with very small proteins because the size of the protein is identical whether it is in the cytoplasm or the lysosomal environment. As a proof of concept, we took advantage of this feature of the dFP reporter to monitor autophagy of mitochondria (mitophagy).

**Figure 6:**
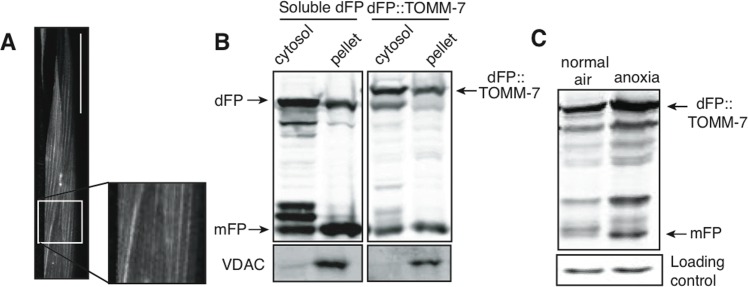
Mitochondrial and cytoplasmic flux through the autophagic pathway (**A**) The mitochondrial outer membrane protein TOMM-7 tagged with dFP was incorporated into mitochondrial patterns in the muscles. Scale bar 50mm. (**B**) Cells from animals expressing either soluble dFP or dFP::TOMM-7 were subfractionated into cytoplasmic and mitochondria-enriched fractions. The presence of VDAC in the pellet confirms mitochondrial enrichment (lower panel). Full-length dFP::TOMM-7 is enriched in the mitochondrial fraction as compared to soluble dFP. (**C**) Animals expressing dFP::TOMM-7 exposed to anoxia showed an increase in mFP release, indicating that autophagy brings mitochondria to the lysosome in response to anoxic shock.

We fused dFP to the outer mitochondrial protein TOMM-7 to measure turnover of mitochondria by mitophagy. TOMM-7 is the *C. elegans* orthologue of Tom7, a key member of the mitochondrial import protein complex [43]. The dFP::TOMM-7 reporter was expressed in all tissues under control of the *eft-3* promoter. Visualization of the fluorescent protein in the body-wall muscle was consistent with its localization to mitochondria (Figure 6A). We confirmed this observation using subcellular fractionation. As expected, dFP::TOMM-7 was more efficiently retained in the mitochondrially-enriched membrane fraction than was the soluble dFP control (Figure 6B). Probing with an antibody against the mitochondrial voltage-dependent anion channel protein (VDAC) confirms that our fractionation effectively concentrated mitochondria. Although most of the soluble dFP was in the cytoplasmic fraction, some soluble dFP (and released mFP) were observed in the high-speed spin pellet. This likely reflects that fact that some lysosomes were included in pellet fraction after this high-speed spin. Consistent with this interpretation, we also observed some free mFP in the pellet from lysate of animals expressing dFP::TOMM-7, though less than is present from animals expressing soluble dFP. We conclude that dFP::TOMM-7 is correctly targeted to mitochondria.

We measured mitophagy in animals exposed to anoxia using the dFP::TOMM-7 reporter. The amount of mFP released was increased in animals exposed to anoxia, similar to what we measured using the dFP::LGG-1 reporter (Figure 6C). This result suggests that mitochondria are degraded by autophagy in anoxia. These studies show the potential power of using the dFP tag to assay the flux of specific proteins and organelles through the autophagic pathway in different tissues.

### Autophagy and lysosomal function in ageing

Genetic data have consistently shown that autophagy is required for increased lifespan in a variety of genetic models [44]. However, it is technically challenging to quantify the effect of normal aging on autophagy in *C. elegans.* Autofluorescence increases with age, particularly in the intestine, making it difficult or impossible to count GFP::LGG-1 punctae. However, the level of autophagic activity changes as animals age, and autophagy genes are required for increased lifespan in many genetic long-lived models [44, 45].

**Figure 7:**
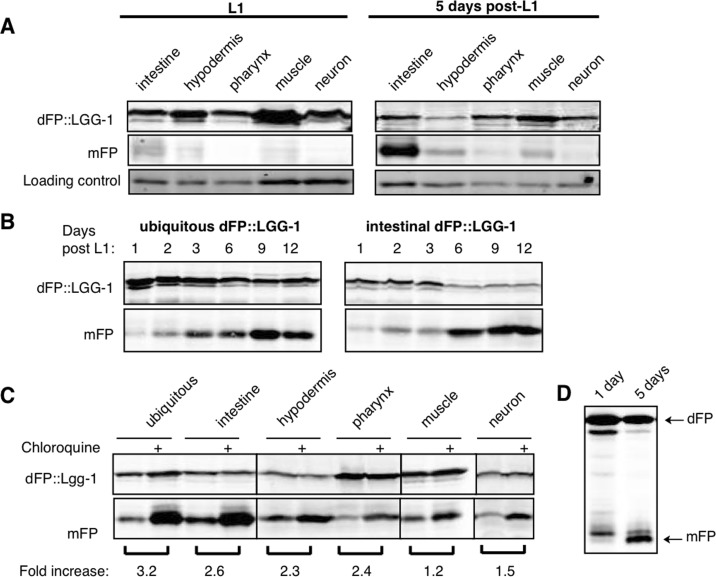
Autophagic flux increases with age (**A**) L1 animals have low levels of mFP in all tissues (left panel), while animals aged to five days after L1 show an increase in mFP in most tissues (right panel, quantifications shown in Supplementary Table 1). Representative blots (of n=4 experiments) are shown. (B) Animals expressing either ubiquitous or intestinal dFP::LGG-1 show a shift in autophagy that occurs in early adulthood, with a dramatic increase in the amount of mFP accumulation between three and six days of life. (**C**) Animals aged five days and treated with chloroquine for 18 hours accumulate more mFP in all tissues, showing that mFP accumulation is not due to a dramatic change in lysosome function. All lanes are from the same experiment run in the same SDS-PAGE gel, with delineations indicating areas of the image that were separately adjusted for brightness and contrast for the sake of clarity (uncropped immunoblots are shown in Supplementary Figure 1). (**D**) Release of mFP from soluble dFP reporter also shows a shift in mFP accumulation with age, with increasing amounts of mFP at five days of age.

We measured the release of mFP from dFP::LGG-1 expressed in different tissues in aging *C. elegans* to test the hypothesis that autophagic flux changes with age in a tissue-specific pattern. We observed an increase in soluble mFP as animals aged, though the effect in pharynx and neurons was not as dramatic as in other tissues (Figure 7A). The overall expression decreased from some promoters in older animals, such as in the hypodermis, but the ratiometric nature of our assay still allowed a measurement of autophagic flux. This result demonstrated an age-dependent increase in autophagic flux that varied between tissues. To determine more precisely when during aging the increase in autophagic flux occurred, we measured steady-state levels of mFP in animals for 12 days after L1 using both ubiquitously-expressed and intestinally-expressed dFP::LGG-1. The ratio of mFP to full-length dFP::LGG-1 increased dramatically in early adulthood, at three to six days after L1 (Figure 7B). Importantly, chloroquine treatment increased the amount of mFP in all tissues (Figure 7C), demonstrating that lysosomes of older animals remain acidified and proteolytically proficient. Moreover, the increase in autophagy measured here is not influenced by the overexpression of LGG-1, as we observed an age-related increase in mFP release from the soluble dFP bulk autophagy reporter (Figure 7D). Thus, our results strongly support the hypothesis that autophagy increases over the course of normal aging.

## DISCUSSION

Autophagy is a powerful but double-edged sword. Depending on context and magnitude, it can promote either cell survival or death, with profound consequences for both normal and disease-state physiology [16, 46–48]. One key to understanding these apparent contradictions at the organismal level may be clarifying the cellular and tissue-specific contexts of autophagy's regulation. Our experiments show that *C. elegans* tissues have dramatically different rates of autophagic flux, and that tissues vary in the degree to which they upregulate autophagy in response to stress and aging. The autophagy map generated here provides a new perspective on the roles of autophagy and TORC1 signaling during development and stress responses.

At a technical level, the dFP assay contributes two key advances to studies of autophagy in *C. elegans.* First, the immunoblot-based assay quantifies the release of mFP at the final step of autophagy, fusion with the lysosome, rather than quantifying the upstream step of autophagosome biogenesis (Figure 1B). This focuses the measure of autophagy on flux through the pathway rather than on fluorescent aggregations that may or may not correspond to productive autophagic structures, and that are obscured by autofluorescence as the animals age [25, 26]. As an immunoblot-based assay the dFP::LGG-1 reporter has the significant benefit of being internally controlled, in that the quantitation of significance is the ratio of full-length to monomeric FP, eliminating the variables of gel loading and protein expression levels that would confound measurement of a single protein band. Second, the validation of the dFP as a soluble or mitochondrially-tagged reporter provides a window onto autophagy in the absence of artificially overexpressed autophagy-related proteins and allows us to monitor specific cargo as it moves through the autophagic pathway. The assay addresses some of the significant limitations of the more commonly-used, microscopy-based assays in *C. elegans* [30, 31].

Our comparison of autophagic flux across tissues showed the highest levels of mFP release at steady-state in the intestine. These results suggest that autophagy may have a unique role or functional significance in the intestine, a conclusion supported by literature showing that perturbation of autophagy in the intestines can have organism-wide effects on lifespan [32, 49]. Autophagy also has a conserved role as a primary line of defense against the invading intracellular pathogenic bacteria in the gut [50–52]. These functions might explain why basal autophagic flux is relatively higher in the intestines than in other tissues. Importantly, the elevated level of mFP in the intestines is seen in lysate from animals with either highly expressed arrays or low expression, single-copy chromosomal insertions (compare Figure 2B and 2C and quantifications in Supplementary Table 1). This demonstrates that the assay is immune to the effects of dFP::LGG-1 protein expression level and thus that results of the assay are not significantly affected by the fact that the tissue-specific promoters have some variation in the amount of protein expression they drive.

Previous studies in *C. elegans* generally have not directly compared autophagic rates between tissues, due to the quantitative and qualitative limitations of morphology-based assessments [20, 22, 31]. For the ubiquitin-proteasome system, the other major degradative pathway involved in proteostasis, there is a quantitative, tissue-specific assay of the degradative processes based on photoconversion. This assay in *C. elegans* revealed substantial mechanistic and functional variation across tissues, with more rapid proteasomal protein turnover in neurons versus body wall muscle [53]. This is in line with our basic observation that tissues have non-uniform rates of protein turnover. Although we did not see dramatic accumulation of mFP in neurons we could detect autophagy in those cells, especially when the hydrolytic enzymes of the lysosome were less active as a result of chloroquine treatment (Figure 3A and Supplementary Table 1). These results are consistent with a situation in which lysosome proteolysis quickly degrades mFP in neurons, which complicates detection of autophagy with the dFP marker. This suggests the interesting possibility that neuronal lysosomes are particularly well poised for protein turnover. Additional quantitative studies will be necessary to understand this phenomenon, and to define how different degradative pathways collaborate to establish and maintain proteostasis in specific tissues.

The differences in autophagic profiles during starvation and anoxia indicate tissue-specific responses to these stresses. Upregulating autophagy is a highly-conserved response to starvation and nutrient deprivation in yeast, nematode and mammalian cells (Figure 1C) [16, 20, 24, 54]. A descriptive, tissue-specific analysis of autophagy in response to starvation in mice found autophagy occurring in almost all tissues [22]. Consistent with this, our assay demonstrated increased autophagy in across tissues in *C. elegans*. These data suggest that autophagy is similarly regulated during nutrient stress in all animals.

Another stimulus that can result in increased autophagy decreased oxygen tension, as occurs in ischemia and reperfusion [48]. Our *C. elegans* model provides a unique animal model of oxygen deprivation. We can precisely manipulate oxygen availability of all cells by changing the external gaseous environment [55, 56]. Autophagy is essential for *C. elegans* survival of anoxia [8]. Our results show that autophagy increases in anoxia, similar to what has been observed in mammalian cells cultured in low-oxygen conditions [57–59]. Autophagy was most strongly up-regulated in the intestine of animals exposed to anoxia.

The different responses to starvation and anoxia were somewhat unexpected, since we chose the duration and severity of the treatments to physiologically stress the animals to roughly similar degrees, as estimated by survival levels [8, 16]. Our results therefore suggest that autophagy has divergent, and divergently regulated, functions across different stress responses, and that it is more responsive in the intestine than in other tissues. The intestine is a major tissue for energy storage, perhaps indicating that, in *C. elegans*, metabolic adaptations to anoxia occur primarily in the intestine.

In contrast to the relatively acute starvation and anoxia stress, accumulation of damage during aging is a chronic homeostatic stress. The aging-associated declines in cellular and organismal function are significantly affected by TORC1 signaling and autophagy. Autophagy is generally thought to counteract the effects of age because inhibiting autophagy causes phenotypes similar to premature aging (and abrogates increased lifespan in many long-lived *C. elegans* backgrounds, including *daf-2* mutant animals), while increasing autophagy delays aging and increases lifespan [3, 19, 60–62].

Our data suggest that there is a change in lysosomal function and autophagic rate that results in an increase in mFP during normal aging. Using chloroquine to inhibit lysosomal proteolysis further increased mFP accumulation above the basal age-associated increase, showing that lysosomes in older animals have not lost their pH-dependent proteolytic activity. The decreased magnitude of mFP accumulation after chloroquine treatment in older compared to younger animals (Figures 7C vs. 3A) offers suggestive evidence that lysosomes in older animals may be less affected by chloroquine than those in younger animals. This would be consistent with age-associated lysosomal deacidification or dysfunction [63, 64], but the dose-dependent effects of chloroquine [36] and the increasing permeability of aging nematodes [65] mean that further quantitative work is necessary to determine the effects age on of progression through the autophagy pathway and lysosomal function.

Significantly, our data add support for the conclusion that there is a dramatic shift in proteostasis that occurs in early adult nematodes, and indicate that this shift is reflected in autophagic activity. By the time animals reach mid-adulthood they have a larger burden of aggregated and unfolded proteins than do younger animals [1, 27, 66, 67]. This accumulation is likely a consequence of shifts across the entire landscape of protein synthesis and degradation, including changes in in protein translation, protease activity, and chaperone-based refolding dynamics [68–70]. It may be that the increase in autophagy we observe is a compensatory response to the accumulation of damaged proteins that occurs at this life stage. Understanding the interplay between autophagy and the integrated proteostasis network will undoubtedly provide important insights into this process.

## METHODS

### Cloning

The dual fluorescent protein (dFP) tag was constructed using tags that were variants of Cerulean and Venus fluorescent proteins (gifts of Erik Snapp, [78]) connected by a linker with GPG, Tev and Cathepsin-sensitive sequences (GGACCAGGCGAGA ATTTGTATTTTCAGGGTCGTCTTGTCAAGTTCCTT GTTGGAGGACCAGGC).

For yeast expression, 990bp of the *S. cerevisiae* ATG8 upstream sequence was cloned into pRS415 followed by the dFP tag cassette and then the ATG8 gene sequence followed by 965bp of downstream sequence. For expression in nematodes, dFP was fused to LGG-1 using plasmids constructed using PCR and Gateway cloning technology (Invitrogen). Plasmids with promoter sequences were kindly provided by Dr. Michael Ailion (University of Washington, Department of Biochemistry). The tissue-specific promoters were *eft-3*p (ubiquitous, also known as *eef-1A.1*), [71]*vha-6*p (intestine) [72], *dpy-7*p (hypodermis) [73], *myo-2*p (pharynx) [74], *myo-3*p (muscle) [75] and *rab-3*p (neurons) [76]. The dFP tag was cloned into the pDONR221 vector and the LGG-1 genomic sequence and 300bp of downstream sequence was cloned into pDONR-P2R-P3 vector. Gateway recombination yielded each promoter upstream of the dFP::LGG-1 coding sequence. Plasmid constructs were verified by sequencing.

For the cytoplasmic dFP expression, dFP was cloned behind the *eft-3* promoter and followed by the *let-858* 3′UTR sequence. The *C. elegans lgg-2* and *tomm-7* genes were cloned into the pDONR-P2R-P3 vector with 572bp or 111 bp of 3′ UTR sequence, respectively. These vectors were recombined with the *eft-3* promoter and dFP plasmids described above to generate each dFP fusion protein expression construct.

### Nematode strains

Arrays were made by injecting Unc EG6699 (ttTi5605 II; unc-119(ed3) III; oxEx1578) worms with the expression constructs and either yeast genomic DNA or an empty yeast expression plasmid to total 100 ng/μL. Offspring with arrays showed normal movement and green fluorescence in the target tissues. Worms with a single, chromosomal insertion of dFP-tagged LGG-1 were made following an established Mos1 mediated Single Copy transgene Insertion (MosSCI) protocol [71] Clonal lines were established for all lines except the LGG-2 arrays, which were mixed populations from a single successful injection. Identical experimental results were obtained both with and without outcrossing for all lines. Worms expressing GFP::LGG-1 (adIs2122) were from the Caenorhabditis Genetics Center.

### Imaging

Images were taken using an Olympus Fluoview FV1200 confocal microscope using a 40x objective and a pinhole setting of 115μm. Images are of a single image slice of worms at approximately larval stage 3 visualized with 515nm excitation and 527nm emission. To count dFP::LGG-1 punctae, L3 animals were placed in a Nikon Eclipse 90i microscope, and the punctae visible in hypodermal seam cells through a GFP filter were counted.

### Immunoblots and antibodies

Animals were harvested by centrifugation and lysed in SDS sample buffer by repeated cycles of freezing and boiling, cleared by centrifugation at 8,000 × g for 5 minutes, and the supernatant was run on a 12% electrophoresis polyacrylamide gel containing sodium dodecyl sulfate (SDS-PAGE) and transferred to a nitrocellulose membrane (Bio-Rad Laboratories). The membrane was blocked for one hour in Odyssey blocking buffer (Li-Cor Biosciences). The membrane was incubated with anti-GFP monoclonal antibody (Roche Applied Science), for detecting either FP or GFP, polyclonal anti-actin (gift from William T. Wickner) or anti-VDAC (Pierce) overnight. After washing in PBST buffer (137 mM NaCl, 2.7 mM KCl, 20 mM dibasic sodium phosphate, 2mM monobasic potassium phosphate, 0.1% Tween-20), the membranes were incubated with the species-appropriate, Alexa-conjugated secondary antibody (IR Dye 800 goat anti-mouse from Rockland Immunochemicals or Alexa Fluor 680 goat anti-rabbit from Invitrogen), washed in PBST, and visualized using the Odyssey Imaging System (Li-Cor Biosciences). ImageJ software (National Institutes of Health; http://imagej.nih.gov/ij/index.html) was used for the densitometry calculations, and for statistical comparison we used a two-tailed t-test.

Animal culture and treatments. Worms were grown at 20° on NGM plates with *E. coli* OP50, or on enriched plates seeded with *E. coli* NA22 cells, as indicated. To developmentally synchronize populations, embryos were isolated using a standard bleaching protocol [77]. To synchronize aging populations, isolated embryos were grown to fourth-stage larvae (L4) and then moved to plates containing 50μM 5-Fluoro-2′-deoxyuridine (FuDR) and grown for the indicated number of days.

In starvation experiments, isolated embryos were hatched in M9 without food overnight. The arrested first-stage larvae (L1) were incubated in 3mL of M9, rotating at 20° for a total of 5 days, with one change of M9. A control sample of L1 animals was harvested, lysed, and frozen at the beginning of the starvation period.

For exposure to anoxia (operationally defined as < 10 ppm O2), isolated embryos were placed directly on OP50 bacteria and grown in room air at 20° for approximately 30 hours to stage L3. Animals were then collected in M9 media, washed twice, and placed on unseeded plates at 20° in room air or in chambers continuously flooded with humidified 100% nitrogen (Airgas, Seattle, WA) for 18 hours [55]. Compressed gas tanks were certified standard to contain < 10 ppm O_2_.

For chloroquine treatment, isolated embryos were grown on OP50 bacteria for approximately 55 hours at 20° and then washed and placed into M9 containing 4x concentrated OP50 and 20mM chloroquine diphosphate salt (Sigma-Aldrich) and incubated, rotating, for 18 hours. For chloroquine treating older worms, animals were grown on OP50 from embryo to L4 when they were moved to NA22 plates containing FuDR. Five days after initial isolation the animals were washed off the plates and incubated for 18 hours in M9 medium containing 4x concentrated OP50 bacteria and 50μM FuDR, with or without chloroquine for 18 hours.

### Yeast growth and starvation

BY4742 yeast were transformed with dFP::ATG8 using a lithium acetate transformation protocol [79] Yeast were cultured overnight in synthetic medium lacking leucine to ensure plasmid retention and upon reaching an optical density (OD600nm wavelength) of 0.8 were resuspended in synthetic medium without leucine, or a nitrogen starvation medium (0.175 wt/vol nitrogen base without ammonium sulfate or amino acids and 2% glucose) for the indicated time. Yeast cells were then harvested by pelleting, resuspending in lysis buffer (50mM Hepes pH 7.4, 200mM NaCl, 10% glycerol, 0.5% Triton-X) with SDS PAGE loading buffer (final concentrations of 62mM Tris pH 6.7, 69 mM sodium dodecyl sulfate, 10% glycerol with bromophenol blue) in the presence of protease inhibitors, heated to 100° for 10 minutes, vortexed with glass beads to break apart the cell wall, cleared from the beads, and centrifuged at 16,000 × g for two minutes. The supernatant lysate was separated by SDS-PAGE and analyzed by immunoblot.

### RNAi

*E. coli* expressing *bec-1* RNAi or the empty control plasmid L4440 were struck onto plates containing 50μg/ml carbenicillin and 15μg/ml tetracycline. A single colony from that plate was cultured overnight in ampicillin-containing medium and plated onto NGM-lite plates that contained 3 mM isopropyl β-D-1-thiogalactopyranoside (IPTG) and 50μg/ml carbenicilin. These RNAi plates were allowed to dry overnight and stored at 4°. Animals were placed onto RNAi bacteria as embryos and grown for approximately 48 hours at 20° before harvesting for analysis by immunoblot. For hypodermal punctae counts, animals were placed on RNAi at L4, allowed to lay eggs, and the progeny were grown to L3 before imaging.

## SUPPLEMENTARY INFORMATION, TABLE AND FIGURE



## References

[1] Ben-Zvi A, Miller EA, Morimoto RI (2009). Collapse of proteostasis represents an early molecular event in Caenorhabditis elegans aging. Proc Natl Acad Sci U S A.

[2] Powers ET, Morimoto RI, Dillin A, Kelly JW, Balch WE (2009). Biological and chemical approaches to diseases of proteostasis deficiency. Annu Rev Biochem.

[3] Rubinsztein DC, Mariño G, Kroemer G (2011). Autophagy and aging. Cell.

[4] Tanaka K, Matsuda N (2014). Proteostasis and neurodegeneration: the roles of proteasomal degradation and autophagy. Biochim Biophys Acta.

[5] Kimura N, Tokunaga C, Dalal S, Richardson C, Yoshino K, Hara K (2003). A possible linkage between AMP-activated protein kinase (AMPK) and mammalian target of rapamycin (mTOR) signalling pathway. Genes Cells.

[6] Jia K, Chen D, Riddle DL (2004). The TOR pathway interacts with the insulin signaling pathway to regulate C. elegans larval development, metabolism and life span. Development.

[7] Meissner B, Boll M, Daniel H, Baumeister R (2004). Deletion of the intestinal peptide transporter affects insulin and TOR signaling in Caenorhabditis elegans. J Biol Chem.

[8] Samokhvalov V, Scott BA, Crowder CM (2008). Autophagy protects against hypoxic injury in C. elegans. Autophagy.

[9] Sancak Y, Peterson TR, Shaul YD, Lindquist RA, Thoreen CC, Bar-Peled L, Sabatini DM (2008). The Rag GTPases bind raptor and mediate amino acid signaling to mTORC1. Science.

[10] Kovacs AL, Zhang H (2010). Role of autophagy in Caenorhabditis elegans. FEBS Lett.

[11] Wang K, Klionsky DJ (2011). Mitochondria removal by autophagy. Autophagy.

[12] Murrow L, Debnath J (2013). Autophagy as a stress-response and quality-control mechanism: implications for cell injury and human disease. Annu Rev Pathol.

[13] Yang P, Zhang H (2014). You are what you eat: multifaceted functions of autophagy during C. elegans development. Cell research.

[14] Meléndez A, Tallóczy Z, Seaman M, Eskelinen E-L, Hall DH, Levine B (2003). Autophagy genes are essential for dauer development and life-span extension in C. elegans. Science.

[15] Mizushima N, Yoshimori T, Ohsumi Y (2011). The role of Atg proteins in autophagosome formation. Annual review of cell and developmental biology.

[16] Kang C, You YJ, Avery L (2007). Dual roles of autophagy in the survival of Caenorhabditis elegans during starvation. Genes & development.

[17] Boya P, Reggiori F, Codogno P (2013). Emerging regulation and functions of autophagy. Nat Cell Biol.

[18] Choi AM, Ryter SW, Levine B (2013). Autophagy in human health and disease. New England Journal of Medicine.

[19] Zoncu R, Efeyan A, Sabatini DM (2010). mTOR: from growth signal integration to cancer, diabetes and ageing. Nature Reviews Molecular Cell Biology.

[20] Klionsky DJ, Cuervo AM, Seglen PO (2007). Methods for monitoring autophagy from yeast to human. Autophagy.

[21] Klionsky DJ, Abdalla FC, Abeliovich H, Abraham RT, Acevedo-Arozena A, Adeli K (2012). Guidelines for the use and interpretation of assays for monitoring autophagy. Autophagy.

[22] Mizushima N, Yamamoto A, Matsui M, Yoshimori T, Ohsumi Y (2004). In vivo analysis of autophagy in response to nutrient starvation using transgenic mice expressing a fluorescent autophagosome marker. Molecular biology of the cell.

[23] Kang C, Avery L (2009). Systemic regulation of starvation response in Caenorhabditis elegans. Genes Dev.

[24] Hansen M, Chandra A, Mitic LL, Onken B, Driscoll M, Kenyon C (2008). A role for autophagy in the extension of lifespan by dietary restriction in C. elegans. PLoS Genet.

[25] Kuma A, Matsui M, Mizushima N (2007). LC3, an autophagosome marker, can be incorporated into protein aggregates independent of autophagy: caution in the interpretation of LC3 localization. Autophagy.

[26] Wang L, Chen M, Yang J, Zhang Z (2013). LC3 fluorescent puncta in autophagosomes or in protein aggregates can be distinguished by FRAP analysis in living cells. Autophagy.

[27] Gerstbrein B, Stamatas G, Kollias N, Driscoll M (2005). In vivo spectrofluorimetry reveals endogenous biomarkers that report healthspan and dietary restriction in Caenorhabditis elegans. Aging Cell.

[28] Kimura S, Noda T, Yoshimori T (2007). Dissection of the autophagosome maturation process by a novel reporter protein, tandem fluorescent-tagged LC3. Autophagy.

[29] Young B, Wightman R, Blanvillain R, Purcel SB, Gallois P (2010). pH-sensitivity of YFP provides an intracellular indicator of programmed cell death. Plant Methods.

[30] Jenzer C, Simionato E, Legouis R (2015). Tools and methods to analyze autophagy in C. elegans. Methods.

[31] Zhang H, Chang JT, Guo B, Hansen M, Jia K, Kovács AL (2015). Guidelines for monitoring autophagy in Caenorhabditis elegans. Autophagy.

[32] Lapierre LR, Gelino S, Meléndez A, Hansen M (2011). Autophagy and lipid metabolism coordinately modulate life span in germline-less C. elegans. Curr Biol.

[33] Cheong H, Yorimitsu T, Reggiori F, Legakis JE, Wang CW, Klionsky DJ (2005). Atg17 regulates the magnitude of the autophagic response. Molecular biology of the cell.

[34] Shintani T, Klionsky DJ (2004). Cargo proteins facilitate the formation of transport vesicles in the cytoplasm to vacuole targeting pathway. J Biol Chem.

[35] Kim J, Huang WP, Klionsky DJ (2001). Membrane recruitment of Aut7p in the autophagy and cytoplasm to vacuole targeting pathways requires Aut1p, Aut2p, and the autophagy conjugation complex. J Cell Biol.

[36] Ni HM, Bockus A, Wozniak AL, Jones K, Weinman S, Yin XM, Ding WX (2011). Dissecting the dynamic turnover of GFP-LC3 in the autolysosome. Autophagy.

[37] Manil-Ségalen M, Lefebvre C, Jenzer C, Trichet M, Boulogne C, Satiat-Jeunemaitre B, Legouis R (2014). The C. elegans LC3 Acts Downstream of GABARAP to Degrade Autophagosomes by Interacting with the HOPS Subunit VPS39. Dev Cell.

[38] Djeddi A, Michelet X, Culetto E, Alberti A, Barois N, Legouis R (2012). Induction of autophagy in ESCRT mutants is an adaptive response for cell survival in C. elegans. J Cell Sci.

[39] Cann GM, Guignabert C, Ying L, Deshpande N, Bekker JM, Wang L (2008). Developmental expression of LC3alpha and beta: absence of fibronectin or autophagy phenotype in LC3beta knockout mice. Dev Dyn.

[40] Maruyama Y, Sou Y-S, Kageyama S, Takahashi T, Ueno T, Tanaka K (2014). LC3B is indispensable for selective autophagy of p62 but not basal autophagy. Biochemical and biophysical research communications.

[41] von Muhlinen N, Akutsu M, Ravenhill BJ, Foeglein Á, Bloor S, Rutherford TJ (2012). LC3C, bound selectively by a noncanonical LIR motif in NDP52, is required for antibacterial autophagy. Mol Cell.

[42] Alberti A, Michelet X, Djeddi A, Legouis R (2010). The autophagosomal protein LGG-2 acts synergistically with LGG-1 in dauer formation and longevity in C. elegans. Autophagy.

[43] Curran SP, Leverich EP, Koehler CM, Larsen PL (2004). Defective mitochondrial protein translocation precludes normal Caenorhabditis elegans development. J Biol Chem.

[44] Madeo F, Tavernarakis N, Kroemer G (2010). Can autophagy promote longevity?. Nat Cell Biol.

[45] Gelino S, Hansen M (2012). Autophagy - An Emerging Anti-Aging Mechanism. J Clin Exp Pathol.

[46] Erdélyi P, Borsos E, Takács-Vellai K, Kovács T, Kovács AL, Sigmond T (2011). Shared developmental roles and transcriptional control of autophagy and apoptosis in Caenorhabditis elegans. J Cell Sci.

[47] Shintani T, Klionsky DJ (2004). Autophagy in health and disease: a double-edged sword. Science.

[48] Matsui Y, Takagi H, Qu X, Abdellatif M, Sakoda H, Asano T (2007). Distinct roles of autophagy in the heart during ischemia and reperfusion: roles of AMP-activated protein kinase and Beclin 1 in mediating autophagy. Circ Res.

[49] Wang MC, O'Rourke EJ, Ruvkun G (2008). Fat metabolism links germline stem cells and longevity in C. elegans. Science.

[50] Bakowski MA, Desjardins CA, Smelkinson MG, Dunbar TA, Lopez-Moyado IF, Rifkin SA (2014). Ubiquitin-Mediated Response to Microsporidia and Virus Infection in C. elegans. PLoS pathogens.

[51] Benjamin JL, Sumpter R, Levine B, Hooper LV (2013). Intestinal epithelial autophagy is essential for host defense against invasive bacteria. Cell Host Microbe.

[52] Curt A, Zhang J, Minnerly J, Jia K (2014). Intestinal autophagy activity is essential for host defense against Salmonella typhimurium infection in Caenorhabditis elegans. Dev Comp Immunol.

[53] Hamer G, Matilainen O, Holmberg CI (2010). A photoconvertible reporter of the ubiquitin-proteasome system in vivo. Nat Methods.

[54] Lum JJ, DeBerardinis RJ, Thompson CB (2005). Autophagy in metazoans: cell survival in the land of plenty. Nature Reviews Molecular Cell Biology.

[55] Fawcett EM, Horsman JW, Miller DL (2011). Creating defined gaseous environments to study the effects of hypoxia on C. elegans. Journal of visualized experiments: JoVE.

[56] Powell-Coffman JA (2010). Hypoxia signaling and resistance in <i>C elegans</i>. Trends in Endocrinology & Metabolism.

[57] Bellot G, Garcia-Medina R, Gounon P, Chiche J, Roux D, Pouysségur J, Mazure NM (2009). Hypoxia-induced autophagy is mediated through hypoxia-inducible factor induction of BNIP3 and BNIP3L via their BH3 domains. Mol Cell Biol.

[58] Papandreou I, Lim AL, Laderoute K, Denko NC (2008). Hypoxia signals autophagy in tumor cells via AMPK activity, independent of HIF-1, BNIP3, and BNIP3L. Cell Death Differ.

[59] Zhang H, Bosch-Marce M, Shimoda LA, Tan YS, Baek JH, Wesley JB (2008). Mitochondrial autophagy is an HIF-1-dependent adaptive metabolic response to hypoxia. J Biol Chem.

[60] Bjedov I, Toivonen JM, Kerr F, Slack C, Jacobson J, Foley A, Partridge L (2010). Mechanisms of life span extension by rapamycin in the fruit fly Drosophila melanogaster. Cell Metab.

[61] He LQ, Lu JH, Yue ZY (2013). Autophagy in ageing and ageing-associated diseases. Acta Pharmacol Sin.

[62] Vellai T, Takacs-Vellai K, Zhang Y, Kovacs AL, Orosz L, Müller F (2003). Influence of TOR kinase on lifespan in C. elegans. Nature.

[63] Cuervo AM, Dice JF (2000). When lysosomes get old. Experimental gerontology.

[64] Hughes AL, Gottschling DE (2012). An early age increase in vacuolar pH limits mitochondrial function and lifespan in yeast. Nature.

[65] Chisholm AD, Xu S (2012). The Caenorhabditis elegans epidermis as a model skin. II: differentiation and physiological roles. Wiley Interdiscip Rev Dev Biol.

[66] Labbadia J, Morimoto RI (2014). Proteostasis and longevity: when does aging really begin?. F1000Prime Rep.

[67] David DC, Ollikainen N, Trinidad JC, Cary MP, Burlingame AL, Kenyon C (2010). Widespread protein aggregation as an inherent part of aging in C. elegans. PLoS biology.

[68] Kirstein-Miles J, Scior A, Deuerling E, Morimoto RI (2013). The nascent polypeptide-associated complex is a key regulator of proteostasis. EMBO J.

[69] Sarkis GJ, Ashcom JD, Hawdon JM, Jacobson LA (1988). Decline in protease activities with age in the nematode Caenorhabditis elegans. Mech Ageing Dev.

[70] Kern A, Ackermann B, Clement AM, Duerk H, Behl C (2010). HSF1-controlled and age-associated chaperone capacity in neurons and muscle cells of C. elegans. PLoS One.

[71] Frøkjær-Jensen C, Davis MW, Ailion M, Jorgensen EM (2012). Improved Mos1-mediated transgenesis in C. elegans. Nat Methods.

[72] Oka T, Toyomura T, Honjo K, Wada Y, Futai M (2001). Four subunit a isoforms of Caenorhabditis elegans vacuolar H+-ATPase. Cell-specific expression during development. J Biol Chem.

[73] McMahon L, Muriel JM, Roberts B, Quinn M, Johnstone IL (2003). Two sets of interacting collagens form functionally distinct substructures within a Caenorhabditis elegans extracellular matrix. Molecular biology of the cell.

[74] Jantsch-Plunger V, Fire A (1994). Combinatorial structure of a body muscle-specific transcriptional enhancer in Caenorhabditis elegans. J Biol Chem.

[75] Dupuy D, Bertin N, Hidalgo CA, Venkatesan K, Tu D, Lee D (2007). Genome-scale analysis of in vivo spatiotemporal promoter activity in Caenorhabditis elegans. Nat Biotechnol.

[76] Sieburth D, Ch'ng Q, Dybbs M, Tavazoie M, Kennedy S, Wang D (2005). Systematic analysis of genes required for synapse structure and function. Nature.

[77] Porta-de-la-Riva M, Fontrodona L, Villanueva A, Cerón J (2012). Basic Caenorhabditis elegans methods: synchronization and observation. J Vis Exp.

[78] Costantini L, Baloban M, Markwardt M, Guo F, Rizzo M, Verkhusha V (2015). A palette of fluorescent proteins optimized for diverse cellular environments. Nature Comm.

[79] Adams A, Gottschling DE, Kaiser CA, Stearns T (1998). Methods in yeast genetics: a Cold Spring Harbor Laboratory course manual.

