# Supplementary material for: Tissue-specific autophagy responses to aging and stress in C. elegans
Source: Aging (Albany NY). 2015 Jun 30;7(6):419–32. doi: 10.18632/aging.100765 (PMC4505168; doi:10.18632/aging.100765)
Supplement: Supplementary file 1 [file aging-07-0419-s001.pdf]

SUPPLEMENTARY INFORMATION

Supplementary Table 1. Quantification of all bands in all immunoblots included in figures. Included are the densitometric measurements for the upper dFP and lower mFP bands, as well as the ratio between them (lower/upper). Blots from (A) Figure 1, (B) Figure 2, (C) Figure 3, (D) Figure 4 and (E) Figure 7.

A

Figure 1C	control	2 hours	4 hours
upper	82.07	77.40	48.15
lower	35.86	106.75	159.86
ratio	0.44	1.38	3.32

Figure 1E	dFP control	dFP + RNAi	GFP control	GFP + RNAi
upper	76.18	52.47	127.71	75.68
lower	85.98	30.10	105.76	15.04
ratio	1.13	0.57	0.83	0.20

B

Figure 2B	Ubiquitous	Intestine	Hypodermis	Pharynx	Muscle	Neuron
upper	166.80	132.86	168.39	83.65	180.26	65.58
lower	104.15	83.66	52.95	6.80	23.01	15.27
ratio	0.62	0.63	0.31	0.08	0.13	0.23

Figure 2C	Ubiquitous	Intestine	Hypodermis	Pharynx	Muscle	Neuron
upper	192.38	149.04	201.86	115.33	208.36	104.51
lower	150.27	89.05	67.10	4.54	20.16	11.74
ratio	0.78	0.60	0.33	0.04	0.10	0.11

Figure 2D	Ubiquitous	Intestine	Neuron
upper	131.04	114.39	99.92
lower	129.42	121.28	15.49
ratio	0.99	1.06	0.16

C

Figure 3A	Ubiquitous		Intestine		Hypodermis		Pharynx		Muscle		Neuron	
	control	chloro	control	chloro	control	chloro	control	chloro	control	chloro	control	chloro
upper	11880.55	7899.57	5543.53	3127.32	7743.19	6145.74	5449.37	5785.67	7406.39	8683.04	2929.83	3136.25
lower	2681.68	10769.98	2583.23	5624.95	2827.13	6759.72	264.58	950.36	1286.06	2818.49	606.67	2019.54
ratio	0.23	1.36	0.47	1.80	0.37	1.10	0.05	0.16	0.17	0.32	0.21	0.64
fold change		6.04		3.86		3.01		3.38		1.87		3.11

D Figure 4A

Fed	Ubiquitous	Intestine	Hypodermis	Pharynx	Muscle	Neuron
upper	106.36	55.68	51.19	74.14	108.89	73.92
lower	145.35	65.79	66.34	15.74	39.49	57.44
ratio	1.37	1.18	1.30	0.21	0.36	0.78

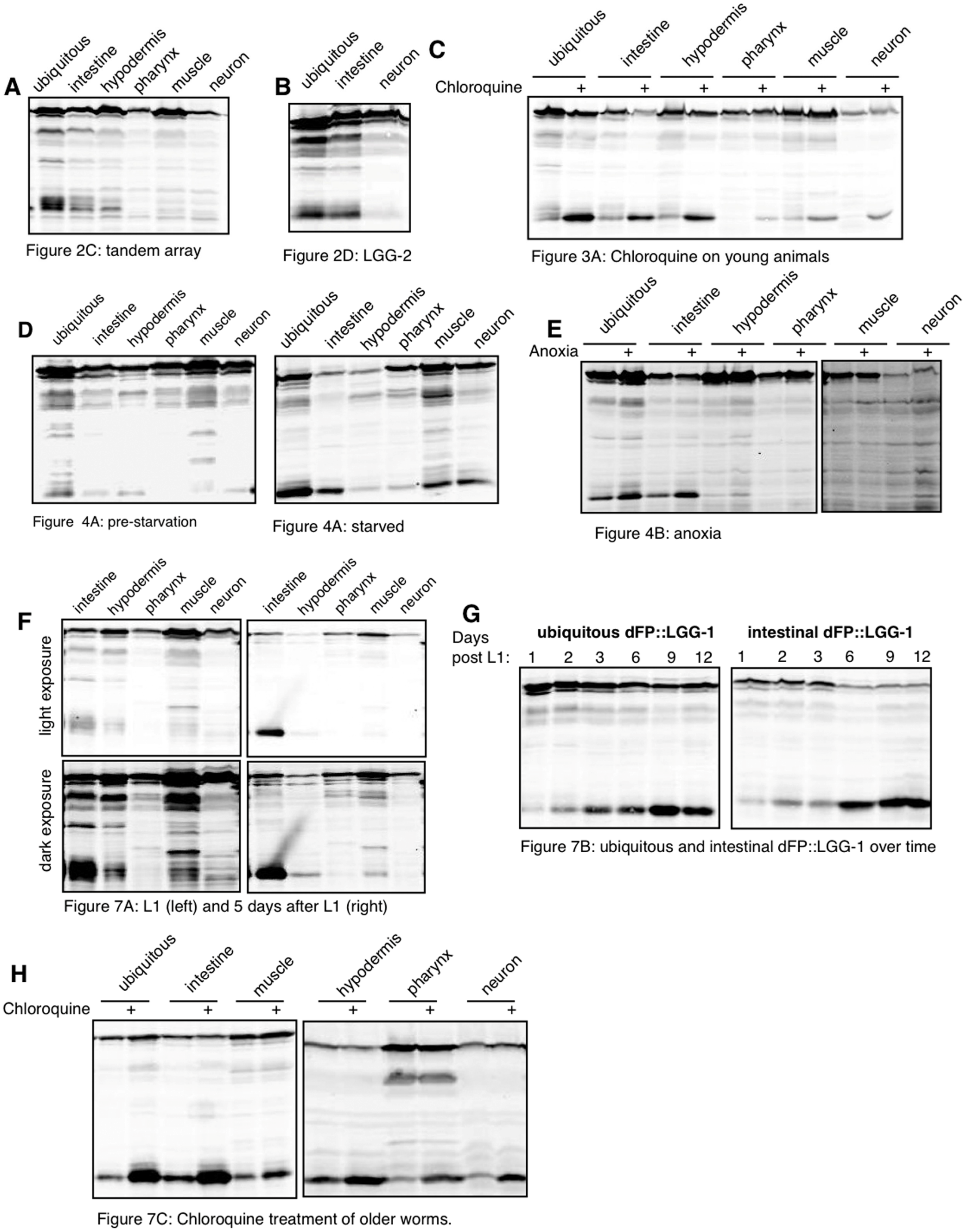

Supplementary Figure 1. Uncropped immunoblots from all figures in which the full-size blot is not shown with figures included in the main body of the text. (**A**,**B**) Immunoblots in Figure 2, (**C**) Figure 3, (**D**, **E**) Figure 4, and (**F**, **G**, **H**) Figure 7. In (**H**) Please note that the order of tissues is altered compared to the original figure in order to show the uncropped image.

.
